# Optic Nerve Changes Detected with Ocular Ultrasonography during Different Surgical Procedures: A Narrative Review

**DOI:** 10.3390/jcm11185467

**Published:** 2022-09-16

**Authors:** Maddalena De Bernardo, Livio Vitiello, Martina De Luca, Aniello La Marca, Nicola Rosa

**Affiliations:** Eye Unit, Department of Medicine, Surgery and Dentistry, “Scuola Medica Salernitana”, University of Salerno, 84081 Salerno, Italy

**Keywords:** optic nerve, optic nerve sheath diameter, ONSD, surgery, ultrasonography

## Abstract

Ultrasonographic appraisal of the optic nerve sheath diameter has become popular in recent years as a useful diagnostic tool to detect intracranial pressure variations. Intracranial hypertension is a life-threatening disease with possible poor clinical outcomes and can be caused by a variety of neurological and non-neurological conditions. Considering the latter, increases in intracranial pressure have also been described during several surgical procedures. Ocular ultrasonography might be utilized to identify intracranial pressure increases by evaluating optic nerve sheath diameter variations. The aim of this review is to provide a wide overview on the use of the optic nerve ultrasound evaluation to detect intracranial pressure changes during surgical procedures, also discussing the pitfalls of the B-scan technique, the most widely used for such a purpose. PubMed medical database, Web of Science and Scopus were used to carry out this review. The present review showed that ocular ultrasonography could be considered a valuable diagnostic tool in the surgical setting to indirectly assess intracranial pressure. However, the use of the B-scan ultrasound should always be coupled with the standardized A-scan technique for a more accurate, precise and trustworthy ultrasound assessment.

## 1. Introduction

Anatomically, the optic nerve attaches to the globe posteriorly and is wrapped in a sheath that contains fluid, with a constant communication between the subarachnoid space of the optic nerve sheath and the intracranial cavity [1]. In fact, as the intracranial pressure (ICP) rises, it also causes an optic nerve sheath diameter (ONSD) increase [2]. The ONSD could be evaluated and measured using ultrasound, as previously described by Ossoinig in the 70s [3,4]. Indeed, due to its anatomic structure, ultrasound ONSD measurement could be considered a useful tool for monitoring ICP in several conditions, also during surgical procedures, in which ICP rise was described as a possible complication [5].

Ocular ultrasonography is a simpler and less invasive diagnostic method compared to other ICP measuring tools [6]. Moreover, ONSD changes could be observed at any time during surgical procedures, and during the follow up [7].

Particularly, standardized ocular echography utilizes specially designed, highly sophisticated real-time A-scan technology, which allows easy and reliable extraction of quantitative data and eliminates falsification of such data by otherwise unavoidable and in part prohibitive artefacts [3,4]. Thus, the ocular ultrasound is standardized, providing a single optimal ultrasonic language and invaluable, understandable, comparable and repeatable results [3,4].

Considering the growing interest in this diagnostic method and its possible application in the surgical setting, the purpose of this review is to identify which surgical procedures could limit significant ICP changes, and how assessment with ocular ultrasonography impacts that objective. This was achieved through the analysis of the published literature in the last 32 years, analyzing all the possible conditions and surgical procedures in which ONSD ultrasound could be considered a useful diagnostic tool to monitor ICP changes. For such a purpose, the first step should be to correlate the ONSD ultrasound measurement with a gold-standard procedure to assess ICP, such as extraventricular drainage.

## 2. Literature Search

PubMed medical database, Web of Science and Scopus were used to search for full articles, case reports, and case series of ultrasound ONSD measurements for the detection of ICP variations during different surgical procedures. Furthermore, additional entries were found by manually searching the references from the original searches.

The earliest date of publication was chosen for January 1990, and the search was completed in June 2022.

## 3. Results

### 3.1. ONSD Ultrasound Evaluation during Extraventricular Drainage

Several papers discussed the ONSD reference values in association with extraventricular drainage to detect intracranial hypertension, with a good sensitivity and specificity, using B-scan ultrasonography [8,9,10,11,12]. However, very different and contrasting ONSD measurements were found in the various papers when ICP exceeded 20 mmHg [8,9,10,11], without a general consensus being reached. In fact, the proposed ONSD cut-off values ranged from 4.80 to 7.14 mm [8,9,10,11], showing how it could be considered challenging to define precise, reliable and repeatable ultrasound values.

Furthermore, some authors also tried to correlate the ONSD/eyeball transverse diameter ratio, and transorbital Doppler ultrasonography parameters with ICP via extraventricular drainage [12]. They found ONSD excluding and including the dura mater and the ONSD/eyeball transverse diameter ratio to be diagnostic parameters significantly associated with ICP, with the latter showing the highest predictive power of increased ICP [12]. Contrariwise, it seems that none of the Doppler ultrasonography parameters were associated with ICP [12].

### 3.2. ONSD Ultrasound Evaluation of the Anaesthetic Effects in Surgical Procedures

In the literature, only three papers analyzed the anaesthetic effects on the ONSD evaluated with ocular ultrasound.

Cennamo et al. [13] measured ONSD with Standardized A-scan in 21 patients to compare two different anesthesiologic techniques. ONSD increase in patients with premedication of atropine and diazepam in combination with intravenously administered ketamine was detected, but not in the group premedicated with fentanyl + droperidol and atropine followed by thiopental and succinylcholine. This could be related to ketamine, which activates the cerebral metabolism, increasing the flow over the barrier as well as the ICP and, consequently, ONSD. For this reason, they suggested to specify the type of anesthetic used in the case of ONSD measurements performed under general anesthesia.

Sujata et al. [14], in 50 patients candidates of propofol- versus sevoflurane-maintained anesthesia in robot-assisted laparoscopic pelvic surgery, found a positive correlation between the duration of surgery and the maximum increase in ONSD in the sevoflurane group but not in the propofol group. They deduced that propofol inhalation attenuated the ICP increase more than sevoflurane.

Geng et al. [15] compared the effect of propofol versus sevoflurane on the ONSD in 110 patients undergoing laparoscopic gynecological surgery, with an estimated operative time of more than 2 h in general anesthesia. Contrary to Sujata et al. [14], the propofol group had ONSD slightly larger than the sevoflurane group, but only in the first 45 min, and not at 1 h after surgery.

Finally, these papers confirm the awareness of Cennamo et al. [13] that researchers should consider which type of anesthesia and which drugs are used due to the possible and very variable effects on the ONSD.

### 3.3. ONSD Ultrasound Evaluation in RALRP

Different studies were carried out on the ONSD ultrasound evaluation with B-scan during RALRP [16,17,18,19,20,21,22,23], showing an increasing trend of ONSD during this surgical procedure. As for the correlation with extraventricular drainage [8,9,10,11,12], also in this case the various papers found very different ONSD ultrasound measurements, with no consensus on the reference value that could indicate the presence of intracranial hypertension. In fact, the ONSD cut-off values widely ranged from 4.30 to 6.80 mm [16,17,18,19,20,21,22,23].

The authors hypothesized different causes to explain this ONSD increase during RALRP, mentioning carbon dioxide pneumoperitoneum and steep Trendelenburg positioning among the main trigger factors [16,17,18,19,20,21,22,23].

In particular, Verdonck et al. [18] supposed that ONSD increased only after depletion of the protective cerebrospinal fluid displacement mechanism, while Shah et al. [19] suggested that increasing venous congestion in steep Trendelenburg position could result in cerebral edema with consequent raised ICP and ONSD increases. On the contrary, Chen et al. [20] considered an internal jugular vein valve incompetency in elderly patients the possible cause of such ONSD increase during RALRP.

### 3.4. ONSD Ultrasound Evaluation during Other Laparoscopic Procedures

Several studies also investigated the ultrasound ONSD variations during other laparoscopic procedures [24,25,26,27,28,29,30], mostly confirming what was hypothesized during RALRP [16,17,18,19,20,21,22,23]. In particular, it seems that ONSD increases during laparoscopic procedures are greater in obese patients [24,25]. This could be related to an acutely elevated intra-abdominal pressure which is able to increase intrathoracic pressure, reducing venous outflow from the central nervous system, thus causing an ICP increase [25].

Furthermore, many authors suggested to use ONSD ultrasound examination to monitor ICP increases during laparoscopic cholecystectomy [26,27,28,29]. In fact, this ultrasound parameter was found to be particularly sensitive in detecting ICP modifications, which occurs when the maximum level of CO_2_ insufflation is reached to create an adequate pneumoperitoneum [26,27,28,29]. This could happen because the intracavity pressures increase and obstruct the venous drainage, with a cerebrospinal liquid absorption reduction, causing an ICP rise with a subsequent ONSD enlargement [28].

As for RALRP and laparoscopic cholecystectomy, Trendelenburg position and pneumoperitoneum were demonstrated to be the main triggers of ICP variations also in the case of laparoscopic hysterectomy [30]. Equally for the previously described laparoscopic procedures, ONSD ultrasound measurements are well correlated with ICP changes [30]. For this reason, transorbital sonography was suggested as a reliable method to monitor intraoperative changes in ONSD, focusing on the need for careful training and the importance of standardization in order to obtain reliable results in the examination technique of ONSD measurements [30].

### 3.5. ONSD Ultrasound Evaluation during Other Surgical Procedures

Other non-laparoscopic surgical procedures were also considered to be at risk of causing intracranial hypertension, showing an ONSD increase detected by ocular ultrasound [31,32,33,34,35,36,37,38,39,40,41,42,43]. Among these, cervical sympathetic block and cervical interlaminar epidural injection were demonstrated to cause ONSD changes related to a possible ICP rise [31,32]. However, despite the risk of intracranial hypertension in these surgical procedures, no increase in ICP-related complications, such as dizziness, headache, visual acuity, or retinal hemorrhage, was observed [32].

Some authors also highlighted how ONSD measurement could be considered a valuable monitoring tool in patients who otherwise would have had no ICP monitoring, as in the case of orthotopic liver transplantation [33]. In fact, ocular ultrasonography could be utilized in the different operative steps to monitor ONSD variations related to possible ICP changes, allowing physicians to have an additional parameter for monitoring the clinical conditions of patients in surgical conditions in which invasive ICP monitoring procedures could not be used [33,34].

Equally, ONSD ultrasound evaluation could be utilized in many other surgical settings, such as spine surgery [35], hemicraniectomy [36], elective tumor craniotomy [37], epiduroscopy [38,39], percutaneous tracheostomy [40] and other elective surgical procedures [41,42,43], always demonstrating a good sensitivity and specificity. It can also be used in pediatric surgery [41,43]. In addition, this ocular ultrasonography showed a good correlation in diagnosing intracranial hypertension compared to other clinical and radiological parameters [37].

However, in all these surgical settings, the reference values found for ONSD are highly variable, most likely due to the absence of a standardization of the most frequently utilized ultrasound techniques and the enormous diversity of surgical interventions in terms of operating techniques and duration procedures. For this reason, the need to develop separate normative values based on the type of surgery may be crucial [43].

## 4. Discussion

Recently, due to its speed of execution, non-invasiveness, and rapidly obtainable results helpful in the diagnosis of several diseases [3,4], ocular ultrasonography has been increasingly popular. This diagnostic method was found to be useful in detecting ICP modifications during surgical procedures in all of the studies included in this review.

In all the discussed papers, except for the paper by Cennamo et al. [13], the distance between the exterior edges of the hypoechogenic subarachnoid space, which is bordered by the dura mater (hyperechogenic) and periorbital fat, was measured at 3 mm posteriorly to the retina, near the edge of the optic nerve sheath.

The use of B-scan sonography is the most often used approach in clinical and surgical setting, but it is well recognized that it has significant drawbacks [44,45,46]. Among the others, the “blooming effect”, resulting in a change in the size of measured structures depending on the sensitivity settings, there is a possibility of having an error in ONSD evaluation with ultrasound, especially utilizing the B-scan approach [47,48,49].

The Standardized A-scan approach, on the other hand, is unaffected by this “blooming effect”, allowing for more exact ONSD measurements thanks to the visible high-reflecting arachnoid spikes [50,51].

Although the Standardized A-scan technique has been already demonstrated by Ossoinig in the 1970s [3,4] and despite being a reliable and effective diagnostic tool in the optic nerve evaluation, in scientific and medical databases, there are rarely papers which utilize this trustworthy technique for this kind of assessment. In fact, in the present review, only the paper by Cennamo et al. [13] discussed the use of A-scan ultrasonography. They measured the ONSD with the Standardized A-scan in 21 subjects to observe how different anesthetic procedures affected the ICP. According to the authors, premedication of atropine and diazepam in association with intravenous ketamine resulted in a considerable ONSD rise. As a result, they concluded that if biometry of the optic nerve is carried out under general anesthesia the type of anesthetic should be stated.

**Figure 1:**
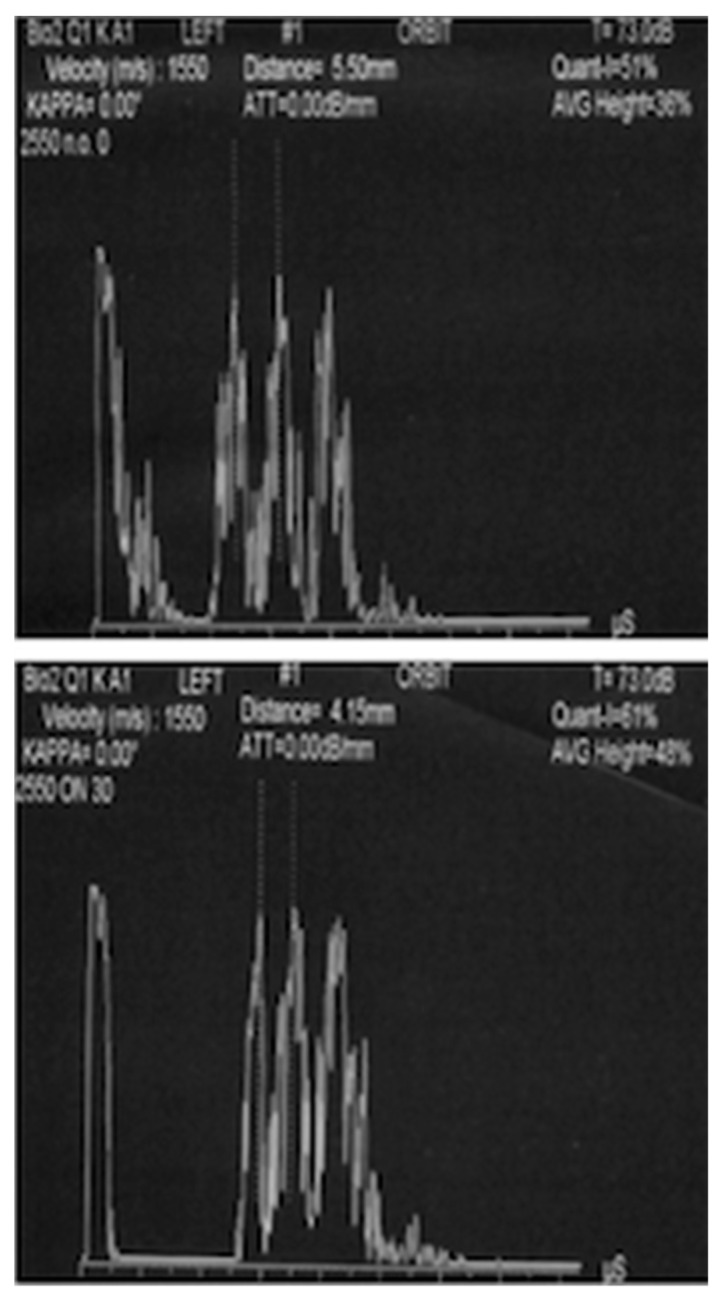
Standardized A-scan image of the optic nerve before (5.50 mm) and after (4.15 mm) “30 degrees test”.

Moreover, the Standardized A-scan allows the use of the “30 degrees test”, a dynamic A-scan test that assesses the width of the optic nerve in primary gaze conditions and again when the patient shifts the gaze temporally [52,53]. In the case of elevated ICP, the nerve and sheaths are stretched, resulting in a smaller diameter (Figure 1).

Solid lesions too can cause the ONSD to appear larger, but the “30 degrees test” will not change the measurement [54,55]. However, this is a more difficult examination that needs a high level of knowledge, and not all ultrasound machines are technologically capable [56,57,58].

Furthermore, it is also possible to perform the “optic nerve exercise” to exclude an optic nerve compartment syndrome [50]. This test is performed by inviting the patient to look alternatingly to the extremely right and left lateral sides for 15 to 20 seconds. The test will lasts three minutes; afterwards, the patient is given three minutes to rest with closed eyes. This allows the subarachnoidal fluid, which is shifted from the orbit during the exercise, to return into the orbit [50]. In healthy people, the original amount of orbital subarachnoidal fluid will be normally restored while; in the case of an optic nerve compartment syndrome, this will not happen [50].

In addition, it is important to point out that, during the ultrasound evaluation in ophthalmology, it is not possible to exactly define the direction of the gaze of a patient with closed eyes. For this reason, the B- or A-scan probe should be utilized with open lids, using methylcellulose and anesthetic drops [56]. This approach allows the eye position to be unmistakably visualized, making the probe orientation much more reliable and avoiding mistakes in detecting gaze direction [56].

## 5. Conclusions

In conclusion, ocular ultrasonography has the advantage of being a non-invasive method of detecting elevated ICP with great sensitivity, even during surgical procedures. Nonetheless, it is crucial to note that combining B-scan ultrasound examinations with the Standardized A-scan method may be more advantageous and accurate in providing the best possible results [59,60].
